# Modeling of three dimensional Prandtl hybrid nano-material over a heated rotating cone involving hall and ion slip currents via finite element procedure

**DOI:** 10.1038/s41598-022-16555-y

**Published:** 2022-07-16

**Authors:** Muhammad Sohail, Umar Nazir, Essam R. El-Zahar, Choonkil Park, Kanit Mukdasai, Amjad Iqbal

**Affiliations:** 1 Department of Mathematics, Khwaja Fareed University of Engineering and Information Technology, Rahim Yar Khan, 64200 Pakistan; 2 Department of Applied Mathematics and Statistics, Institute of Space Technology, P.O. Box 2750, Islamabad, 44000 Pakistan; 3 Department of Mathematics, College of Science and Humanities in Al-Kharj, Prince Sattam Bin Abdulaziz University, P.O. Box 83, Al-Kharj, 11942 Saudi Arabia; 4 Department of Basic Engineering Science, Faculty of Engineering, Menoufia University, Shebin El-Kom, 32511 Egypt; 5 Research Institute for Natural Sciences, Hanyang University, Seoul, 04763 Korea; 6 Department of Mathematics, Faculty of Science, Khon Kaen University, Khon Kaen, 40002 Thailand; 7 Department of Materials Technologies, Faculty of Materials Engineering, Silesian University of Technology, 44-100 Gliwice, Poland

**Keywords:** Mathematics and computing, Nanoscience and technology

## Abstract

Flow in a rotating cone for magnetized Prandtl fluid model is inspected in this investigation. The momentum equation of Prandtl model is derived under the consideration of Hall and ion slip effects and heat transport phenomenon is considered with Joule heating and viscous dissipation effects. The model of Hamilton Crosser and Yamada Ota are considered for the empirical relations of nanofluid mixture. The flow presenting expression of Prandtl fluid model with thermal transport is modeled under boundary layer approximation in the form of partial differential equations (PDEs). The derived PDEs have been converted into set of coupled nonlinear ordinary differential equations (ODEs) by engaging an appropriate scaling group transformation and these converted nonlinear set of ODEs have been tackled numerically via finite element scheme (FES). Impact of different emerging parameters has been displayed graphically and the physics behind the observed phenomena is explained in detail. The convergence of FES is established by carrying the grid independent survey. From the performed investigation, it is recorded that the parameters appear due to Hall and Ion slip currents enhance the fluid velocity but the inverse behavior is recorded for temperature profile.

## Introduction

The study of non-Newtonian fluids got much attention due to their applications in numerous field of applied sciences and engineering. An important non-Newtonian model is Prandtl fluid. Due to application in different mechanisms several researchers have worked on it. The constitutive relation for Prandtl model is

1 $$\tau^{*} = a^{*} \left[ {\left( {\frac{\partial u}{{\partial z}}} \right)^{2} + \left( {\frac{\partial v}{{\partial z}}} \right)^{2} } \right]^{{ - \frac{1}{2}}} \sin^{ - 1} \left[ {c^{*} \left( {\left( {\frac{\partial u}{{\partial z}}} \right)^{2} + \left( {\frac{\partial v}{{\partial z}}} \right)^{2} } \right)^{\frac{1}{2}} } \right]$$

where “${\tau }^{*}$” denotes the stress tensor for Prandtl model and “${a}^{*}$, ${c}^{*}$” are material constants. Several researchers have worked on Prandtl model with different effects. For instance, Hamid et al.^1^ studied numerically the time dependent radiated Prandtl model with thermal and solutal transportation obeying slip mechanism. They used finite difference procedure to solve the modeled equations numerically in MAPLE package. They monitored the escalation in fluid velocity, concentration, and temperature fields respectively against unsteadiness parameter. Akbar et al.^2^ worked on peristaltic transport phenomenon in Prandtl model in an asymmetric channel and handled the arising modeled equations via perturbation scheme. They used a numerical procedure as well for the comparison purpose and presented the tabulated study for the authenticity of obtained solution. They reported the excellent agreement in perturbation solution and numerical solution. They found the decrease in pressure rise against fluid parameter and phase difference. Muser^3^ presented the rheology of Prandtl model in different physical effects under different conditions and recorded the nature of Prandtl model in various situations. Garaud^4^ presented an experimental investigation for double diffusion phenomenon under low Prandtl number assumption. Nadeem et al.^5^ analyzed endoscope while studying peristaltic transport in Prandtl model and monitored the numerous patterns of waves. They computed the solution of modeled equations analytically via perturbation tool. Perturbation solution is computed by Munawar^6^ and analyzed the phenomenon of peristaltic transport in a channel. They noticed the decrease in fluid velocity against magnetic parameter.

The transport phenomenon can be enhanced by mixing the nanoparticles in base fluid. The study of nanofluids is important due to their significant applications in different processes. The study of nanofluids is a charming field of research due to huge applications in different industrial processes. Several researchers have considered nanofluids in their research due to their practical applications and usage. For instance, Das et al.^7^ presented a review of nanofluids with their applications focusing on thermal transport. Godson et al.^8^ presented a survey on escalation in heat transfer using nanofluids in different processes. Daungthongsuk and Wongwises^9^ presented experimental and theoretical survey on convection and enhancement in thermal transport utilizing nanofluids mixture. Vanaki et al.^10^ presented a numerical investigation on heat transport in nanofluids with numerous introduced models and discuss about the thermo-physical features. In another exploration, Solangi et al.^11^ worked on a comprehensive review of nanofluid and explored their properties and preparation mechanism. Also, they highlighted the importance and utilization of nanofluids. Mansour et al.^12^ worked on forced convective thermal transportation in nanofluids. Lomascolo et al.^13^ presented an experimental investigation on thermal transportation in nanofluids and presented the different heat transport modes and expressed their significance. Kim et al.^14^ studied the utilization of nanoparticles in different systems and monitored the boiling effect. Fard et al.^15^ presented the CFD analysis for single phase and two phase nanofluid model in a tube and monitored the comparative heating efficiency. The phenomenon of heating is improved by including the carbon nanotubes in nanofluid modelling was reported by Marquis and Chibante^16^. Several important contributions on nanofluid are covered in^17–20^. Ayub et al.^21^ studied cross nanomaterial in the presence of stagnation point flow inserted magnetic field using velocity slip phenomena. Shah et al.^22^ investigated several features regarding Carreau liquid using approach of nanoparticles under influence of magnetic parameter over wedge. Ayub et al.^23^ discussed several thermal aspects of Carreau liquid involving influence of magnetic field. Shah et al.^24^ captured chemical species and heat energy characteristics in Cross nanofluid inserting an impact of magnetic dipole in cylindrical panels. Ayub et al.^25^ discussed thermal features of Cross fluid considering effects of thermal radiation and Lorentz force using 3D rotating disks. Ayub et al.^26^ studied impacts of hybrid nanomaterial using nanoscale energy phenomena in the presence of magnetic field via Lobatto IIIA approach. Shah et al.^27^ estimated heat energy features of cross fluid inserted approach of nanoparticles on heated surface using cubic autocatalysis. Chamkha and Al-Mudhaf^28^ estimated influences of Lorentz force in mass diffusion and thermal energy in the presence of heat source in rotating cone. Takhar et al.^29^ discussed characterizations of time dependent flow using Lorentz force in energy transfer in heating cone. Reddy et al.^30^ captured features based on mass diffusion and thermal energy in the presence of chemical reaction involving suspension of nanoparticles in base fluid past a porous cone. Chamkha^31^ studied influences of Lorentz force using concept of Hall force in energy transfer phenomena over porous plate. Takhar et al.^32^ captured role of mass diffusion and heat energy in moving cylinder considering bouncy force. Relevant studies are mentioned in^33–42^. Parveen et al.^43^ studied an inclination in energy transfer using thermal radiation and Lorentz force occurring hybrid nanomaterial via entropy generation. Shoaib et al.^44^ studied numerical impacts of Lorentz force in 3D flow in the presence of viscous dissipation and hybrid nanomaterial in rotational disk via used Lobatto IIIA approach. Parveen et al.^45^ studied properties of microorganisms in rheology of peristaltic liquid in the occurrence of Joule heating using nanoparticles. Awais et al.^46^ estimated immersion of nanoparticles in bio-convective flow using concept of viscous dissipations and heat immersion. Khan et al.^47^ observed thermal features of second law analysis occurring viscous dissipation and mixed convection flow. Relevant studies are mentioned in^48–54^.

Available literature shows that no study is performed on three dimensional Prandtl model covering Hall effect with Hamilton Crosser and Yamada Ota model by finite element scheme. This attempt fills this gap. This paper is organized in five sections. A comprehensive review of literature is included in “Introduction”, modeling of Prandtl model in rotating frame under numerous important physical effects is covered in section “Development regarding flow analysis” with dimensionless procedure arising nonlinear boundary value problem is tackled in section “Numerical technique”, and the scheme is implemented and explained in detail; obtained graphical and tabular results are analyzed and explained in section “Outcomes and discussion” and important findings are covered in section “Prime consequences”.

## Development regarding flow analysis

Consider the thermal performance of ethylene glycol of 3D Prandtl fluid of nanoparticles and hybrid nanoparticles over a cone rotating with angular velocity. Models regarding Hamilton crosser and Yamada Ota are inserted. The wall temperature is considered to be non-uniform. Further, the effects of Hall, heat source and ion forces are taken to be significant and the concept of buoyancy forces is generated due to force of gravity. The flow of viscous fluid is induced due to rotating of heated cone. *MoS*_*2*_*–SiO*_*2*_-ethylene glycol is considered as plasma and shear thinning. The set of PDEs are obtained using basic laws under boundary layer approximations. The following effects have been considered in PDEs while modeling the flow over a rotating cone.

- Stress tensor of Prandtl fluid is used in desired PDEs;
- The concept of Hall and ion slip forces are considered;
- 3D rotating flow in heated cone is visualized;
- Bouncy force and Lorentz force are addressed;
- Joule heating and heat source are implemented;
- Hamilton Crosser and Yamada Ota models for hybrid nanofluid are considered;
- Thermal properties of ethylene glycol, titanium dioxide and silicone dioxide are assumed.

**Figure 1:**
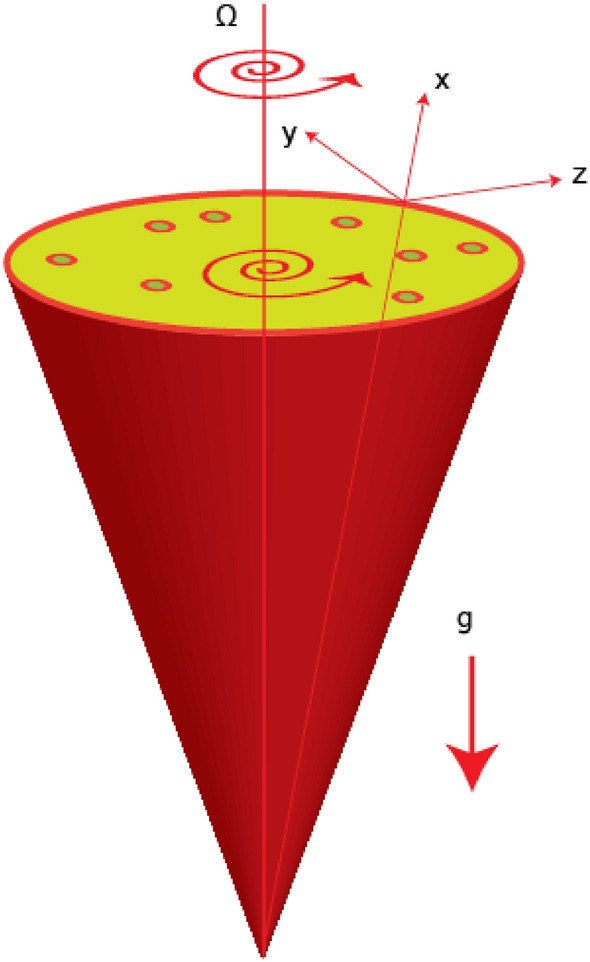
Rotating cone for developed problem.

The direction of gravitational acceleration is considered downward in direction of axis via cone. Z-axis is normal component of cone and motion of particles is rotated with angular velocity $\Omega .$ Temperature at away of wall and for a way of wall is considered as ${T}_{w}$ and ${T}_{\infty }.$ The velocity components $\widetilde{w},\, \widetilde{u}$ and $\widetilde{v}$ along $z,\, x$ and $y-$ directions. The physical flow model is captured by Fig. 1.

2 $$\frac{{\partial \left( {x\tilde{u}} \right)}}{\partial x} + \frac{{\partial \left( {x\tilde{v}} \right)}}{\partial z} = 0,$$

3 $$\begin{aligned} \widetilde{u}\frac{\partial \widetilde{u}}{\partial x}+\widetilde{w}\frac{\partial \widetilde{u}}{\partial z}&=\frac{{\left(\widetilde{v}\right)}^{2}}{x}+{\nu }_{hnf}\left(\frac{A}{C}\frac{{\partial }^{2}\widetilde{u}}{\partial {z}^{2}}+\frac{A}{2{C}^{3}}{\left(\frac{\partial \widetilde{u}}{\partial z}\right)}^{2}\frac{{\partial }^{2}\widetilde{u}}{\partial {z}^{2}}\right)+g\beta \left(T-{T}_{\infty }\right){\mathrm{cos}}\alpha\\ &\quad +\frac{{\left({B}_{0}\right)}^{2}{\sigma }_{hnf}}{{\rho }_{hnf}\left[{\left(1+{\beta }_{i}{\beta }_{e}\right)}^{2}+{\left({\beta }_{e}\right)}^{2}\right]}\left[\widetilde{v}{\beta }_{e}-\left(1+{\beta }_{e}{\beta }_{i}\right)\widetilde{u}\right], \end{aligned}$$

4 $$\begin{aligned} \widetilde{u}\frac{\partial \widetilde{v}}{\partial x}+\widetilde{w}\frac{\partial \widetilde{v}}{\partial z}&=-\frac{\widetilde{v}\widetilde{u}}{x}+{\nu }_{hnf}\left(\frac{A}{C}\frac{{\partial }^{2}\widetilde{v}}{\partial {z}^{2}}+\frac{A}{2{C}^{3}}{\left(\frac{\partial \widetilde{v}}{\partial z}\right)}^{2}\frac{{\partial }^{2}\widetilde{v}}{\partial {z}^{2}}\right)\\ &\quad -\frac{{\left({B}_{0}\right)}^{2}{\sigma }_{hnf}}{{\rho }_{hnf}\left[{\left(1+{\beta }_{i}{\beta }_{e}\right)}^{2}+{\left({\beta }_{e}\right)}^{2}\right]}\left[\widetilde{u}{\beta }_{e}+\left(1+{\beta }_{e}{\beta }_{i}\right)\widetilde{v}\right], \end{aligned}$$

5 $$\begin{aligned} \widetilde{u}\frac{\partial T}{\partial x}+\widetilde{w}\frac{\partial T}{\partial z}&=\frac{{k}_{hnf}}{{\left(\rho {C}_{p}\right)}_{hnf}}\frac{{\partial }^{2}T}{\partial {z}^{2}}+\frac{{{\left({\beta }_{0}\right)}^{2}\sigma }_{hnf}}{{\left(\rho {C}_{p}\right)}_{hnf}\left[{\left(1+{\beta }_{i}{\beta }_{e}\right)}^{2}+{\left({\beta }_{e}\right)}^{2}\right]}\left[{\left(\widetilde{u}\right)}^{2}+{\left(\widetilde{v}\right)}^{2}\right]\\ &\quad +\frac{Q}{{\left(\rho {C}_{p}\right)}_{hnf}}\left(T-{T}_{\infty }\right). \end{aligned}$$

The boundary conditions at wall and away from of wall are generated due to no-slip theory

6 $$\widetilde{u}=0,\,\, T={T}_{w},\,\, \widetilde{v}=\Omega x\,{\mathrm{sin}}\,\alpha ,\,\, \widetilde{u}\to 0,\,\, \widetilde{v}\to 0,\,\, T\to {T}_{\infty }.$$

Transformations are defined as

7 $$\widetilde{u}=-\frac{\Omega x\,{\mathrm{sin}}\,\alpha {f}^{{\prime}}}{2},\,\, \widetilde{v}=\Omega x\,{\mathrm{sin}}\,\alpha g,\,\, \widetilde{w}={\left({\nu }_{f}{\Omega\, {\mathrm{sin}}}\,\alpha \right)}^\frac{1}{2}f,\,\, T={T}_{\infty }+\left({T}_{w}-{T}_{\infty }\right)\theta ,\,\,\eta =z{\left(\frac{{\Omega \,{\mathrm{sin}}}\,\alpha }{{\nu }_{f}}\right)}^\frac{1}{2}.$$

Correlations insight into nanoparticles and hybrid nanoparticles are mentioned below and physical properties are listed in Table 1.

8 $$\left.\begin{array}{l}{\rho }_{hnf}=\left[\left(1-{\varnothing }_{2}\right)\left\{\left(1-{\varnothing }_{1}\right){\rho }_{f}+{\varnothing }_{1}{{\rho }_{s}}_{1}\right\}\right]+{\varnothing }_{2}{{\rho }_{s}}_{2}\\ {\left(\rho {C}_{p}\right)}_{hnf}=\left[\left(1-{\varnothing }_{2}\right)\left\{\begin{array}{c}\left(1-{\varnothing }_{1}\right){\left(\rho {C}_{p}\right)}_{f}\\ +{\varnothing }_{1}{{\left(\rho {C}_{p}\right)}_{s}}_{1}\end{array}\right\}\right] {+\varnothing }_{1}{{\left(\rho {C}_{p}\right)}_{s}}_{2}\\ \left\{\frac{{{k}_{s}}_{1}+\left(n-1\right){k}_{f}-\left(n-1\right){\varnothing }_{1}\left({k}_{f}-{{k}_{s}}_{2}\right)}{{{k}_{s}}_{1}+\left(n-1\right){k}_{f}-{\varnothing }_{1}\left({{k}_{s}}_{2}-{k}_{f}\right)}\right\}=\frac{{k}_{bf}}{{k}_{f}}\end{array}\right\},$$

9 $$\left.\begin{array}{l}{\mu }_{hnf}=\frac{{\left(1-{\varnothing }_{2}\right)}^{2.5}{\mu }_{f}}{{\left(1-{\varnothing }_{1}\right)}^{2.5}},\frac{{k}_{nf}}{{k}_{f}}=\left\{\frac{{k}_{s}+\left(n+1\right){k}_{f}-\left(n-1\right)\varnothing \left({k}_{f}-{k}_{s}\right)}{{k}_{s}+\left(n-1\right){k}_{f}+\varnothing \left({k}_{f}-{k}_{s}\right)}\right\}\\ \frac{{k}_{hnf}}{{k}_{bf}}=\left\{\frac{{{k}_{s}}_{2}+\left(n-1\right){k}_{bf}-\left(1-n\right){\varnothing }_{2}\left({{k}_{s}}_{2}-{k}_{bf}\right)}{{{k}_{s}}_{2}+\left(n-1\right){k}_{bf}-{\varnothing }_{2}\left({k}_{bf}-{{k}_{s}}_{2}\right)}\right\}\\ \left\{\frac{{{k}_{s}}_{2}+\left(n-1\right){k}_{bf}-\left(1-n\right){\phi }_{2}\left({{k}_{s}}_{2}-{k}_{bf}\right)}{{{k}_{s}}_{2}+\left(n-1\right){k}_{bf}-{\phi }_{2}\left({k}_{bf}-{{k}_{s}}_{2}\right)}\right\}=\frac{{k}_{hnf}}{{k}_{bf}}\end{array}\right\},$$

10 $$\left. {\begin{array}{*{20}l} {\frac{{k_{hnf} }}{{k_{bf} }} = \left\{ {\frac{{\frac{{k_{s2} }}{{k_{bf} }} + \omega + \omega \emptyset_{2} \left( {1 - \frac{{k_{s2} }}{{k_{bf} }}} \right)}}{{\frac{{k_{s2} }}{{k_{bf} }} + \omega + \emptyset_{2} \left( {1 - \frac{{k_{s2} }}{{k_{bf} }}} \right)}}} \right\} \omega = 2\emptyset_{2}^{\frac{1}{5}} \frac{L}{D} \text{ for cylindrical particle}} \\ {\omega = 2\emptyset_{2}^{1/5} \text{for spherical particle}} \\ \end{array} } \right\},$$

11 $$\left. {\begin{array}{*{20}l} {\frac{{k_{hnf} }}{{k_{bf} }} = \left\{ {\frac{{\frac{{k_{s1} }}{{k_{f} }} + \omega + \omega \emptyset_{1} \left( {1 - \frac{{k_{s1} }}{{k_{f} }}} \right)}}{{\frac{{k_{s1} }}{{k_{f} }} + \omega + \emptyset_{1} \left( {1 - \frac{{k_{s1} }}{{k_{f} }}} \right)}}} \right\}\omega = 2\emptyset_{2}^{\frac{1}{5}} \frac{L}{D} \,\, \text{for cylindrical particle}} \\ {\omega = 2\emptyset_{2}^{1/5}\,\, \text{for spherical particles}} \\ \end{array} } \right\}.$$

**Table 1:** Thermal properties for density ($\rho$), heat capacity ($C_{p}$) and thermal conductivity ($k$)^12^.

	$k$ (thermal conductivity)	$C_{p}$ (heat capacity)	$\rho$ (density)
$C_{2} H_{6} O_{2}$	0.253	2430	1113.5
$TiO_{2}$	8.4	692	4230
$SiO_{2}$	1.4013	3.5 × 10^6^	2270

Formulated ODEs are

12 $$\begin{aligned} \frac{{\nu_{f} }}{{\nu_{hnf} }}\left( {\frac{1}{2}\left( {f^{\prime}} \right)^{2} - ff^{\prime\prime} - 2g^{2} - 2\lambda \theta } \right) &- \frac{{\left( {1 - \emptyset_{1} } \right)^{2.5} M^{2} \left( {1 - \emptyset_{2} } \right)^{2.5} }}{{\left[ {\left( {1 + \beta_{i} \beta_{e} } \right)^{2} + \left( {\beta_{e} } \right)^{2} } \right]}}\left[ {2\beta_{e} g + \left( {1 + \beta_{i} \beta_{e} } \right)f^{\prime}} \right] \\ &\quad + \alpha f^{{\prime\prime\prime}} + \beta \left( {f^{\prime\prime}} \right)^{2} f^{{\prime\prime\prime}} = 0,\end{aligned}$$

13 $$\begin{aligned} \frac{{\nu_{f} }}{{\nu_{hnf} }}\left( {gf^{\prime} - f^{\prime}g^{\prime}} \right)& - \frac{{\left( {1 - \emptyset_{1} } \right)^{2.5} M^{2} \left( {1 - \emptyset_{2} } \right)^{2.5} }}{{\left[ {\left( {1 + \beta_{i} \beta_{e} } \right)^{2} + \left( {\beta_{e} } \right)^{2} } \right]}}\left[ {\left( {1 + \beta_{i} \beta_{e} } \right)g - \frac{1}{2}\beta_{e} f^{\prime}} \right] \\ &\quad + \alpha g^{^{\prime\prime}} + \beta \left( {g^{\prime}} \right)^{2} g^{^{\prime\prime}} = 0, \end{aligned}$$

14 $$\begin{aligned} &\theta^{\prime\prime} + \frac{{k_{f} }}{{k_{hnf} }}\frac{{\left( {\rho C_{p} } \right)_{hnf} }}{{\left( {\rho C_{p} } \right)_{f} }}Pr\left( {\frac{1}{2}f^{\prime}\theta - f\theta ^{\prime}} \right) + \frac{{k_{f} }}{{k_{hnf} }}\frac{{M^{2} EcPr}}{{\left[ {\left( {1 + \beta_{i} \beta_{e} } \right)^{2} + \left( {\beta_{e} } \right)^{2} } \right]}}\left( {\frac{1}{4}f^{{\prime}{2}} + g^{2} } \right) \\ &\quad + \frac{{k_{f} }}{{k_{hnf} }}H_{s} Pr\theta = 0.\end{aligned}$$

Boundary conditions in term of dimensionless are

15 $$f^{\prime}\left( 0 \right) = 0,\,\, f^{\prime}\left( \infty \right) = 0,\,\, g\left( 0 \right) = 1,\,\, g\left( \infty \right) = 0,\,\, \theta \left( \infty \right) = 0,\,\, f\left( 0 \right) = 0,\,\, \theta \left( 0 \right) = 1.$$

Dimensionless parameters utilized in Eqs. (12) and (14) are defined as

$$\begin{aligned} Pr &= \frac{{\left( {C_{p} } \right)_{f} \mu_{f} }}{{k_{f} }}, M^{2} = \frac{{\left( {\beta_{0} } \right)^{2} \sigma_{nf} }}{{\rho_{f} {\Omega }\sin \alpha_{1} }},Ec = \frac{{\left( {U_{w} } \right)^{2} }}{{\left( {T_{w} - T_{\infty } } \right)\left( {C_{p} } \right)_{f} }} , \lambda = \frac{{gL\beta \cos \alpha_{1} }}{{\left( {\nu_{f} } \right)^{2} {\Omega }\sin \alpha_{1} }},\\ H_{s} &= \frac{Q}{{{\Omega }sin\alpha_{1} \left( {\rho C_{p} } \right)_{f} }}, \alpha = \frac{1}{{A\mu_{f} C}}, \beta = \frac{{{\Omega }\left( {U_{w} } \right)^{2} }}{{2\nu_{f} C^{2} }}. \end{aligned}$$

Divergence of velocity along horizontal and vertical direction is

16 $$\left( {Re} \right)^{\frac{1}{2}} C_{f} = \frac{{ - \left[ {\alpha f^{\prime\prime}\left( 0 \right) + \beta \left( {f^{\prime\prime\prime}\left( 0 \right)} \right)^{3} } \right]}}{{\left( {1 - \emptyset_{1} } \right)^{2.5} \left( {1 - \emptyset_{2} } \right)^{2.5} }},\quad \left( {Re} \right)^{\frac{1}{2}} C_{g} = \frac{{ - \left[ {\alpha g^{\prime}\left( 0 \right) + \beta \left( {g^{\prime\prime}\left( 0 \right)} \right)^{3} } \right]}}{{\left( {1 - \emptyset_{1} } \right)^{2.5} \left( {1 - \emptyset_{2} } \right)^{2.5} }}.$$

The mathematical relation for temperature gradient (Nusselt number) is

17 $$\left( {Re} \right)^{{\frac{ - 1}{2}}} NU = \frac{{ - k_{hnf} }}{{k_{f} }}\theta^{\prime}\left( 0 \right).$$

## Numerical technique

The numerical approach is adopted as a finite element scheme^52^ (FEM)^17,18^ to compute the solution of highly ODEs (non-linear). The domain of current model is broken into small segments called finite element scheme. The applications of FEM are used in electrical systems, solid mechanics, chemical processing and fluid related problems etc. The solution related steps of FEM is listed below.

- Step-I: Weak form is made from strong form (said ODEs) and residuals are formulated;
- Step-II: Shape functions are taken as linearly and Galerkin finite element scheme is utilized to obtain weak form;
- Step-III: Assembly process is utilized to construct stiffness elements and global stiffness matrix is formulated;
- Step-IV: Algebraic system (nonlinear equations) is obtained with help of Picard linearization approach;
- Step-V: Algebraic equations are simulated via 10^−5^ (computational tolerance) using following stopping criteria;
  18 $$\left| {\frac{{X_{i + 1} -X_{i} }}{{X^{i} }}} \right| < 10^{ - 5} .$$
- Step-VI: Table 2 reveals investigation of mesh-free;

**Table 2:** Simulations of mesh free analysis for velocities and temperature when $\emptyset_{1} = 0.002,\emptyset_{2} = 0.0075, \lambda = 0.3, \beta_{e} = 0.3, \alpha = 2.0, \beta_{i} = 0.002, \beta = 0.2, Pr = 206, Ec = 3.2,H_{s} = 0.2, M = 0.01.$

Number of elements	$f^{\prime}\left( {\frac{{\eta_{max} }}{2}} \right)$	$g\left( {\frac{{\eta_{max} }}{2}} \right)$	$\theta \left( {\frac{{\eta_{max} }}{2}} \right)$
30	0.6586386817	0.0001721355025	0.6605821625
60	0.6233792179	0.6437292447	0.0002229082388
90	0.6116725178	0.6380761926	0.0002319259575
120	0.6058311283	0.6352430888	0.0002348216628
150	0.6023296966	0.6335412345	0.0002360353595
180	0.5999974251	0.6324058465	0.0002366270036
210	0.5983324921	0.6315945612	0.0002369433746
240	0.5970846646	0.6309857193	0.0002371243282
270	0.5961135026	0.6305118421	0.0002372274024
300	0.5953371601	0.6301329244	0.0002372898241
- Step-VII: Convergence analysis is confirmed via 300 elements.

### Validation of problem

Code and problem are validated in case of Nusselt number and flow rates with published work. It can be estimated that good agreements are simulated between published work and present problem by disappearing impacts of ion slip, Hall current, heat sink and viscous dissipation in current problem. This validation is recorded in Table 3.

**Table 3:** Validation in view of numerical behavior for flow rates and temperature gradient when $\emptyset_{1} = \emptyset_{2} = 0.0, \beta_{e} = 0.0, \alpha = 2.0, \beta_{i} = 0.0, \beta = 0.2, Pr = 0.7, Ec = 0.0,H_{s} = 0.0.$

$\lambda$	Present work			Malik et al.^54^		
	$- \left( {Re} \right)^{\frac{1}{2}} C_{f}$	$- \left( {Re} \right)^{\frac{1}{2}} C_{g}$	$- \left( {Re} \right)^{{\frac{ - 1}{2}}} NU$	$- \left( {Re} \right)^{\frac{1}{2}} C_{f}$	$- \left( {Re} \right)^{\frac{1}{2}} C_{g}$	$- \left( {Re} \right)^{{\frac{ - 1}{2}}} NU$
0.0	0.6330341643	0.6153201962	0.4295412140	1.0253	0.6153	0.4295
1.0	2.2006812241	0.8492300251	0.6128301282	2.2007	0.8492	0.6121
10	8.5982303219	1.3993024924	1.009332492	8.5041	1.3990	1.0097

## Outcomes and discussion

Modeled equations are solved via FEM and parametric analysis is carried out in order to examine transport of momentum and heat.

### Outcomes of velocity fields

**Figure 2:**
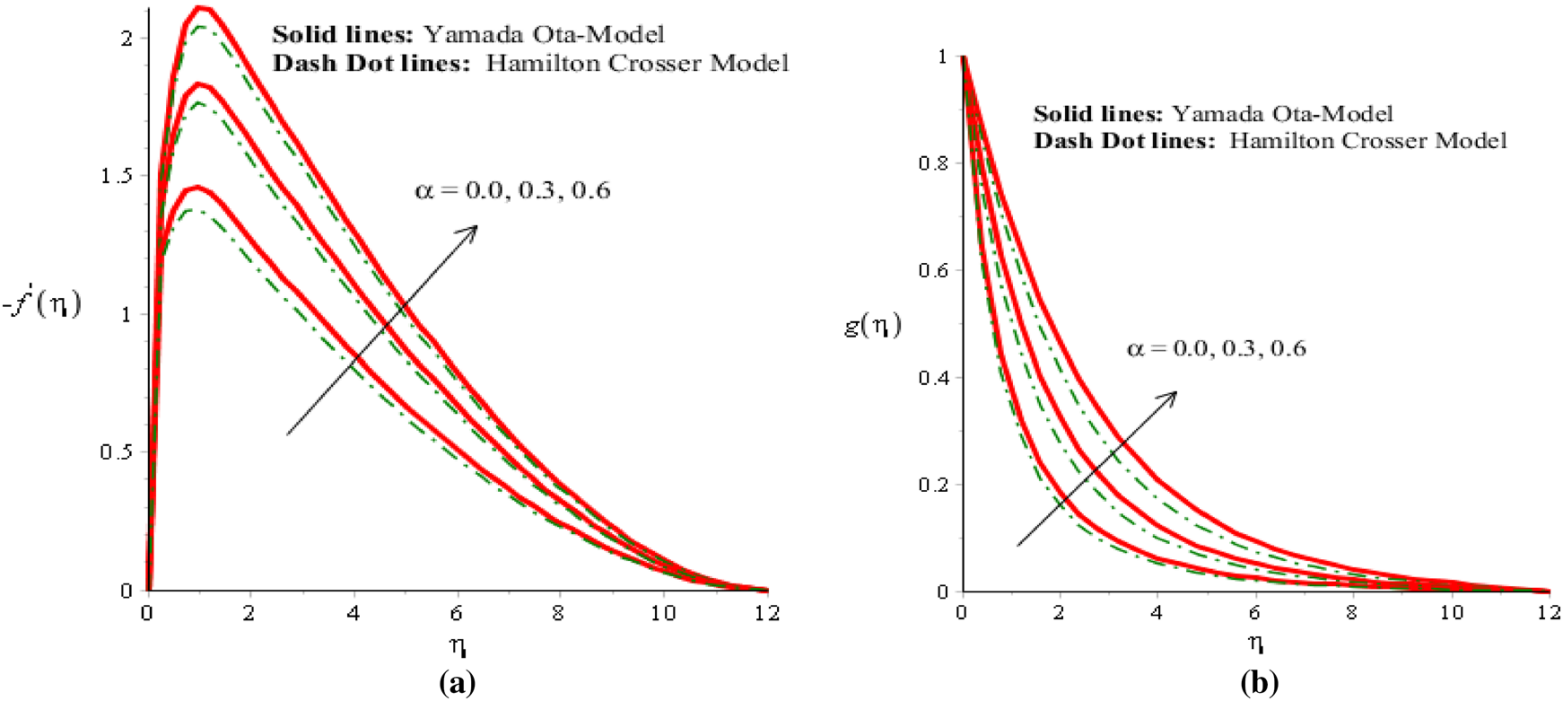
(**a**,**b**) Velocity field in y-direction and x-direction against $\alpha$ when $\emptyset_{1} = 0.002,\emptyset_{2} = 0.0075, \lambda = 2.0, \beta_{e} = 0.2, \beta_{i} = 0.01, \beta = 0.3, Pr = 206, Ec = 2.2,H_{s} = - 0.2, M = 0.03.$

**Figure 3:**
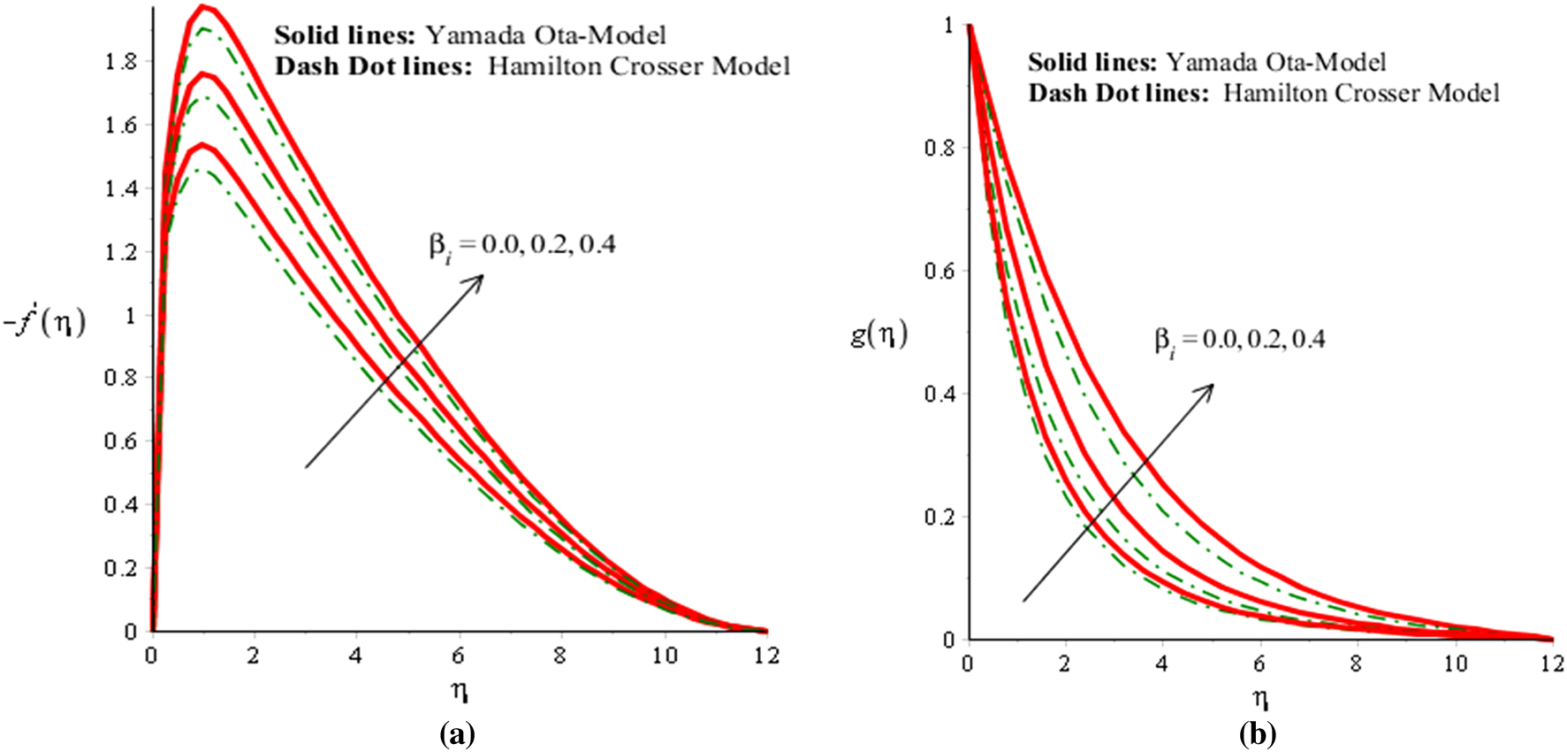
(**a**,**b**) Velocity field in y-direction and x-direction against $\beta_{i}$ when $\emptyset_{1} = 0.002,\emptyset_{2} = 0.0075, \lambda = 0.3, \beta_{e} = 2.0, \alpha = 2.0, \beta = 00.2, Pr = 206, Ec = 3.0,H_{s} = 2.0, M = 0.3.$

**Figure 4:**
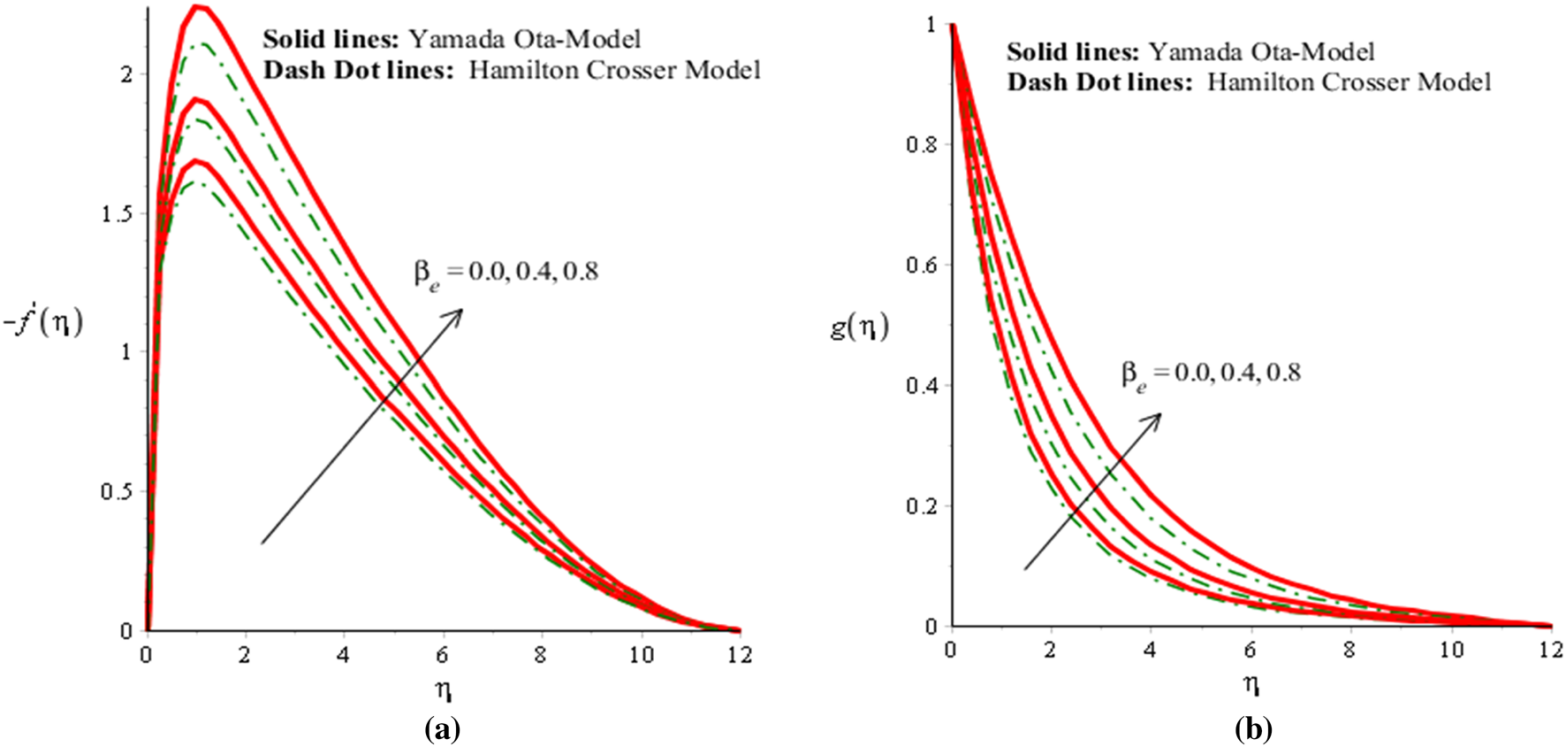
(**a**,**b**) Velocity field in y-direction and x-direction against $\beta_{i}$ when $\emptyset_{1} = 0.002,\emptyset_{2} = 0.0075, \lambda = 2.3, \alpha = 2.0, \beta_{i} = 0.02, \beta = 2, Pr = 206, Ec = 0.1,H_{s} = 0.4, M = 0.3.$

The solid curves are for velocity components of Yamada Ota model whereas dot curves are for velocity components of Hamilton Crosser hybrid nanomaterial model. An influence of $\alpha$ on flow distribution in both components is carried out by Fig. 2a,b. It is noticed that parameter related to $\alpha$ is formulated due to appearance of Prandtl rheology in momentum equations. Mathematically, directly proportional relation is investigated between velocity curves and fluid number. Therefore, flow is produced slowly down versus implication of $\alpha .$ Hence, viscosity of fluid is increased when fluid number is increased. Thickness for momentum layers is also declined against change in $\alpha .$ Flow produced by Yamada Ota model is higher than flow produced by Hamilton Crosser hybrid nanomaterial model. Figures 3a,b, 4a,b reveal influences of ion slip number ($\beta_{i}$) and Hall number ($\beta_{e}$) on flow components in y- and x-directions. It is observed that ion slip and Hall currents are produced using concept of generalized Ohm’s law in current problem. In mathematically, flow distributions have direct proportional relation against $\beta_{e}$ and $\beta_{i} .$ Therefore, argumentation insight into fluidic particles is predicted against implication of $\beta_{e}$ and $\beta_{i} .$ Physically, ($\beta_{i}$) ion slip number is produced by product among ion cyclotron frequency and ion collision time. An increment into ion slip number produces an inclination into ion cyclotron frequency and ion collision time. This physical reason is produced an enhancement into motion regarding both directions. Velocity curves are significantly increased for Yamada Ota model rather than Hamilton Crosser hybrid nanomaterial model. It is noted through the impact of Hall and ion slip currents on velocity in case of ethylene glycol are displayed. The flow of ethylene glycol is funded to be plasma and exposed to be applied magnetic field. Hall and ion forces are generated due to the interaction of plasma and applied magnetic field. The Lorentz force is reduced due to an increase in Hall and ion slip parameters. This fact is because of Hall, ion forces and magnetic field are opposite which are responsible reduction in Lorentz force. The flow is accelerated because of Hall and ion forces for both cases of nanofluids and hybrid nanofluids. Due to opposite behavior among Lorentz force and ion slip and Hall forces, flow was enhanced and momentum thickness was also enhanced when Lorentz force was declined. This impact of Hall and ion slip currents on the flow is observed. Hence, Yamada Ota hybrid nano-metallic particles should be dispersed in pure fluid is recommended for its better thermal performance rather than Hamilton Crosser hybrid model.

### Outcomes of temperature field

**Figure 5:**
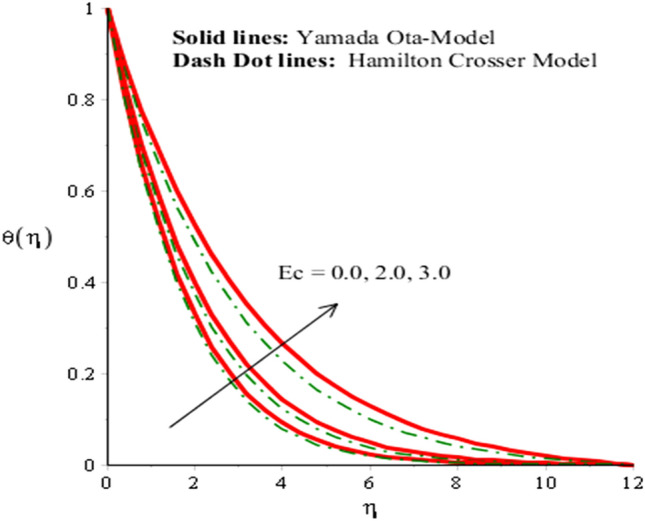
Analysis of heat energy field in against $Ec$ when $\emptyset_{1} = 0.002,\emptyset_{2} = 0.0075, \lambda = 0.3, \beta_{e} = 0.4, \alpha = 3.0, \beta_{i} = 2.0, \beta = 0.2, Pr = 206, H_{s} = - 3.0, M = 0.03.$

**Figure 6:**
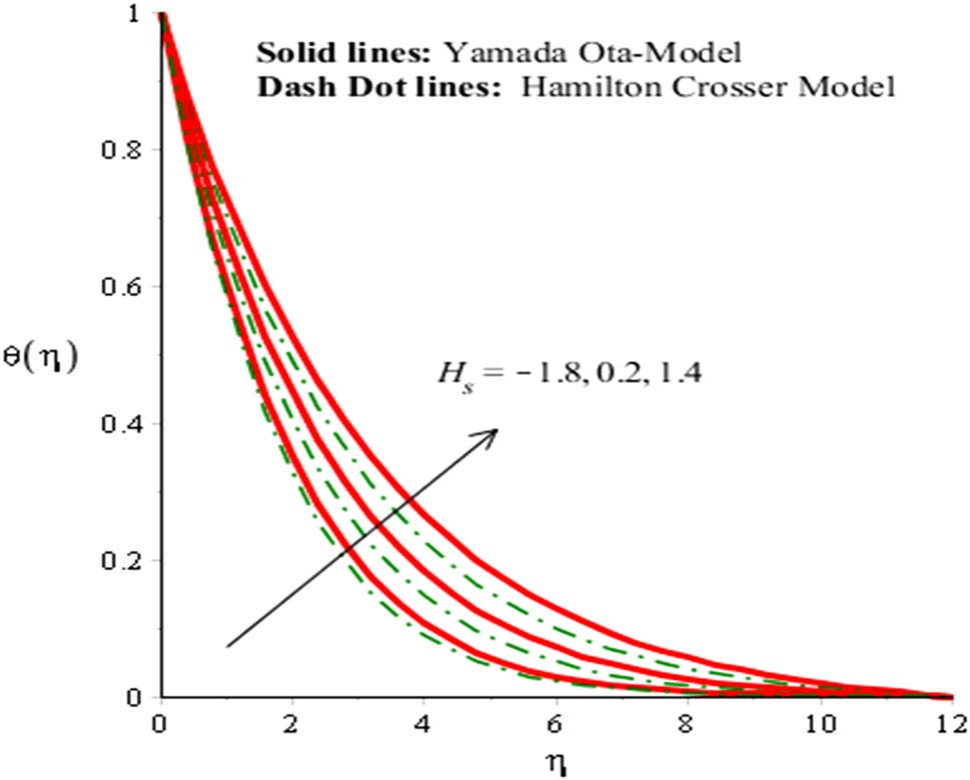
Analysis of heat energy field in against $H_{s}$ when $\emptyset_{1} = 0.002,\emptyset_{2} = 0.0075, \lambda = 0.3, \beta_{e} = 0.3, \alpha = 2.0, \beta_{i} = 0.2, \beta = 0.9, Pr = 206, Ec = 3, M = 0.01.$

**Figure 7:**
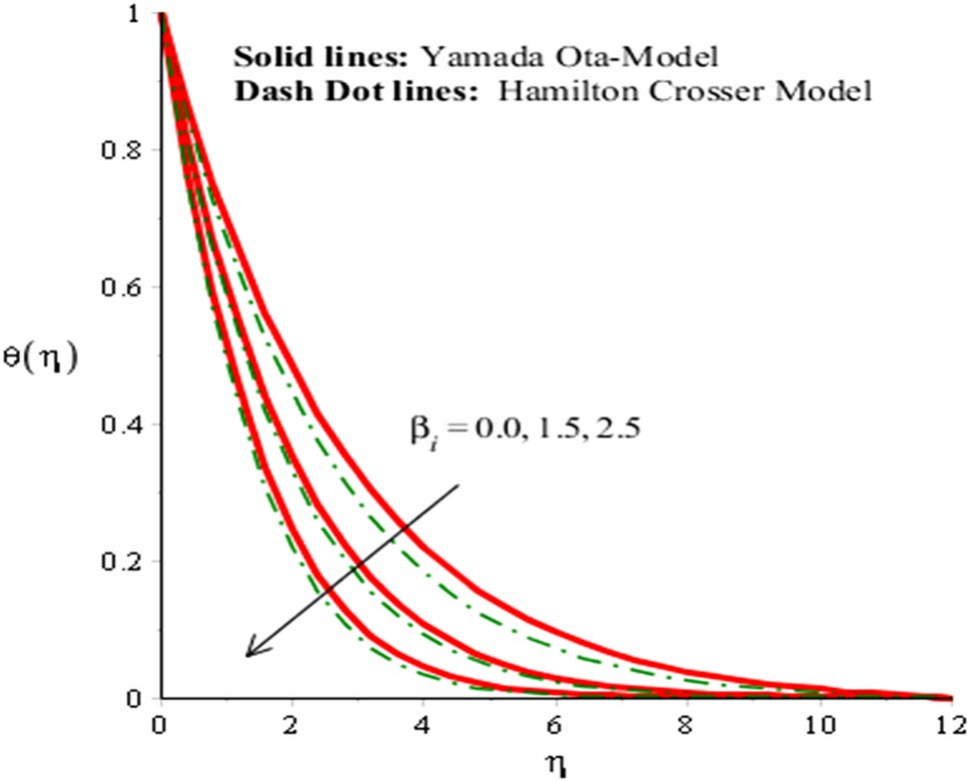
Analysis of heat energy field in against $\beta_{i}$ when $\emptyset_{1} = 0.002,\emptyset_{2} = 0.0075, \lambda = 4.0, \beta_{e} = 0.3, \alpha = 2.0, \beta = 0.2, Pr = 206, Ec = 4.0,H_{s} = 0.2, M = 2.0.$

**Figure 8:**
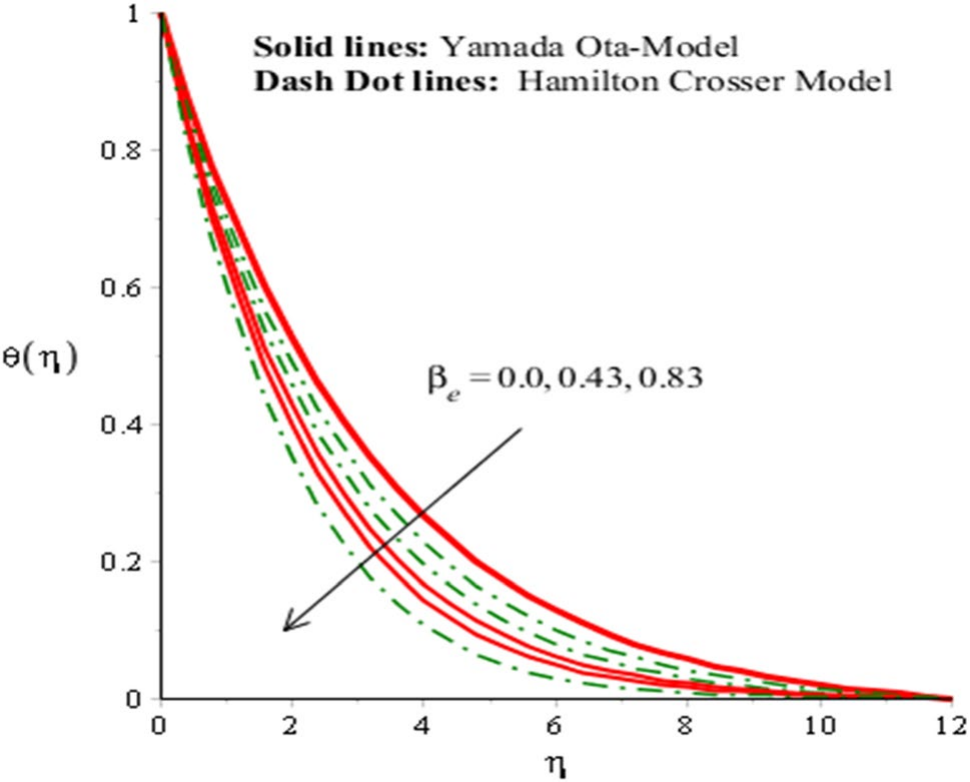
Analysis of heat energy field in against $\beta_{e}$ when $\emptyset_{1} = 0.002,\emptyset_{2} = 0.0075, \lambda = 0.3, \alpha = 2.0, \beta_{i} = 0.002, \beta = 0.8, Pr = 206, Ec = 3.2,H_{s} = 0.2, M = 0.01.$

The solid curves are for velocity components of Yamada Ota model whereas dot curves are for velocity components of Hamilton Crosser hybrid nanomaterial model. It is included that an efficiency of Yamada Ota model increases rather than the ethylene glycol regarding Hamilton Crosser hybrid nanomaterial model. Figure 5 demonstrates impact of Eckert number on thermal distribution. It is noticed that *Ec* is produced using concept of viscous dissipation into energy equation. It is coefficient of joule heat and viscous dissipation. An inclination in *Ec* results heat energy is significantly dissipated because of friction force and Joule heating. Heat energy adds into fluid particles because of frictional force. Hence, temperature is increased. Furthermore, thermal layers thickness for case of Yamada Ota hybrid nanomaterial model is higher than for case of Hamilton Crosser hybrid nanomaterial model. Figure 6 represents role of *H*_*s*_ (heat source number) on thermal profile. Two type’s influences were conducted into fluidic particles which are based on heat absorption and heat generation. External thermal source was placed at wall. So, thermal energy can be managed using variation of *H*_*s*_. Hence, Yamada Ota hybrid nanomaterial model in ethylene glycol is recommended for its better thermal performance rather than Hamilton Crosser hybrid nanomaterial model. Additionally, heat is generated due to enhancement in Eckert number. This fact is due to $Ec$ acts as coefficient of viscous dissipation term. Therefore, more heat is produced due friction between the fluid particles. The role of Hall and ion-slip numbers on the temperature for both cases of two hybrid models in Figs. 7 and 8. However, temperature decreases when ion slip and Hall parameters are increased via Hall and ion slip parameters because Hall and ion slip currents play a key role in a controlling the dissipation of heat energy. Physically, collision into electron and ions are called ion slip and Hall forces. The direction among magnetic field and applied Lorentz force is opposite. Therefore, Lorentz force decreases when $\beta_{i}$ and $\beta_{e}$ are increased. Hence, TBLT (thermal boundary layer thickness) can be shortened via external magnetic field and use fluid in plasma state.

### Investigation regarding skin friction and Nusselt number

The impacts of $M, \beta_{e} , \beta_{i}$ and $H_{s}$ on the behavior skin friction coefficients and heat energy rate is observed and noted numerical values are displayed in Table 4. Magnetic field parameter has shown an increasing impact on wall shear stresses. But heat energy rate was decreased when Lorentz force is significantly enhanced. It is noted through shear stresses increase when $\beta_{e}$ is increased whereas $\beta_{e}$ is responsible for significant decrease in shear stress but more heat generates due to $\beta_{e}$ into particles. Heat flux of mono-nanofluid and hybrid nanofluid decreases when heat source number is enhanced. However, the impact of shear stresses is decreased due to an increase in $H_{s} .$

**Table 4:** Numerical impacts of skin friction coefficients and heat transfer rate versus $\beta_{e} , \beta_{i} , H_{s}$ and $M$ when $\emptyset_{1} = 0.002,\emptyset_{2} = 0.0075, \lambda = 2.0, \beta_{i} = 0.01, \beta = 0.3, Pr = 206, Ec = 2.2.$

Change in parameters		$\left( {Re} \right)^{\frac{1}{2}} C_{f}$	$\left( {Re} \right)^{\frac{1}{2}} C_{g}$	$\left( {Re} \right)^{\frac{1}{2}} NU$
$M$	0.0	0.5553481694	1.748625433	0.7829754254
	0.3	0.6552943007	1.754302238	0.7237240327
	0.6	0.7551674911	1.783088915	0.7161334864
$\beta_{e}$	0.0	0.5553328293	1.748062602	0.7831495083
	0.4	0.4553404122	1.648160325	0.8830709949
	0.8	0.3553430627	1.628288537	0.9830395354
$H_{s}$	-1.8	0.5160815058	1.659381887	0.794817722
	0.4	0.4011296359	1.518494450	0.674217273
	0.8	0.3943834879	1.509256446	0.410370366
$\beta_{i}$	0.0	0.5555095192	1.746942157	0.7606163594
	0.5	0.4555101361	1.647591937	0.8606106152
	1.7	0.3555110713	1.617968095	0.9605989369

## Prime consequences

The ethylene glycol containing hybrid nano-structures subjected to magnetic field helps to use magneto-hydrodynamic equations to model the transportation of momentum and heat. Heat source is accumulated in the presence of Hamilton crosser and Yamada Ota models. Numerical solutions are computed via FEM and key outcomes are recorded which are listed here.

- Hall and ion slip currents help to accelerated flow in both x and y directions. However, temperature decreases when Hall and ion currents are increased because of Hall and ion slip currents play a key role in a controlling the dissipation of heat energy. Hence, the thermal boundary layer thickness can be controlled through the external magnetic field used is in plasma state;
- Hybridity of nano-structures in plasma ethylene glycol exposed to magnetic field. It is noted through extensive numerical experiments that hybrid nano-structures increases the thermal performances of ethylene glycol relative to mono-nano-structures;
- Wall shear stress in x-direction in case of ethylene glycol mono-nanofluid is greater than the wall shear stress in x-direction for the case of hybrid nanofluid and vice versa for wall shear stress in y-direction.

## Data Availability

The datasets generated/produced during and/or analyzed during the current study/research are available from the corresponding author on reasonable request.

## List of symbols

*τ*^***^: Stress tensor (N m^−^^2^)
$$\widetilde{u},\, \widetilde{w},\, \widetilde{v}$$: Velocity components (m s^−^^1^)
Ω: Rotating velocity (m s^−^^1^)
*β*: Thermal expansion coefficient (K^−^^1^)
*T*_∞_: Ambient temperature (K)
*σ*: Electrical conductivity (S m^−^^1^)
*β*_*i*_: Ion slip number (–)
*ρ*: Fluid density (kg m^−^^3^)
*k*: Thermal conductivity (kg s^−^^3^ K^−^^1^ m)
*ϕ*_1_, *ϕ*_2_: Volume fractions for nanoparticles (–)
*β**, **α*: Fluid parameters (–)
*M*: Magnetic number (–)
*H*_*s*_: Heat source number (–)
*TiO*_*2*_: Titanium dioxide (–)
FEM: Finite element method (–)
*Re*: Reynolds number (–)
*C*_*f*_: Skin friction coefficient
PDEs: Partial differential equations
*a*^***^, *c*^***^: Material constants (–)
*x, z*: Space coordinates (m)
*g*: Gravitational acceleration (ms^−^^2^)
*T*: Fluid temperature (K)
*β*_0_: Magnitude of magnetic field (Am^−^^1^)
*β*_*e*_: Hall parameter (–)
$$\nu$$: Kinematic viscosity (m^2^ s^−^^1^)
*C*_*p*_: Specific heat capacity (J kg^−^^1^ K)
*Q*: Heat source (J)
*λ*: Mixed convection number (–)
*Pr*: Prandtl number (–)
*Ec*: Eckert number (–)
*C*_*2*_*H*_*6*_*O*_*2*_: Ethylene glycol (–)
*SiO*_*2*_: Silicon dioxide (–)
$\theta ,\, f,\, g$: Dimensionless temperature and velocities (–)
*NU*: Nusselt number (–)
*η*: Independent variable
ODEs: Ordinary differential equations
